# From PEF to PBF: What difference does the longer alkyl chain make a computational spectroscopy study of poly(butylene 2,5-furandicarboxylate)

**DOI:** 10.3389/fchem.2022.1056286

**Published:** 2022-12-06

**Authors:** Mariela M. Nolasco, Leonor C. Rodrigues, Catarina F. Araújo, Mariana M. Coimbra, Paulo Ribeiro-Claro, Pedro D. Vaz, Svemir Rudić, Armando J. D. Silvestre, Chaima Bouyahya, Mustapha Majdoub, Andreia F. Sousa

**Affiliations:** ^1^ CICECO, Departamento de Química, Universidade de Aveiro, Aveiro, Portugal; ^2^ Champalimaud Foundation, Champalimaud Centre for the Unknown, Lisboa, Portugal; ^3^ ISIS Neutron & Muon Source, STFC Rutherford Appleton Laboratory, Didcot, United Kingdom; ^4^ Laboratoire des Interfaces et Matériaux Avancés, Université de Monastir, Monastir, Tunisia; ^5^ Centre for Mechanical Engineering, Materials and Processes, Department of Chemical Engineering, University of Coimbra, Coimbra, Portugal

**Keywords:** computational spectroscopy, inelastic neutron scattering (INS), C-H...O hydrogen bond, molecular interpretation, physical properties, 2,5-furan dicarboxylate, butylene glycol

## Abstract

This work explores the conformational preferences and the structure-property correlations of poly(butylene 2,5-furandicarboxylate) (PBF), a longer chain analogue of the most well-known biobased polyester from the furan family, poly(ethylene 2,5-furandicarboxylate) (PEF). A thorough computational spectroscopic study–including infrared, Raman and inelastic neutron scattering spectroscopy, combined with discrete and periodic density functional theory calculations–allowed the identification of dominant structural motifs in the amorphous and crystalline regions. Discrete calculations and vibrational spectroscopy of semi-crystalline and amorphous samples strongly support the predominance of *gauche, trans, gauche* conformations of the butylene glycol fragment in both the crystalline and amorphous domains. In what concerns the furandicarboxylate fragment, amorphous domains are dominated by *syn,syn* conformations, while in the crystalline domains the *anti,anti* forms prevail. A possible crystalline structure–built from these conformational preferences and including a network of C-H···O hydrogen bond contacts—was optimized using periodic density functional theory. This proposed crystal structure avoids the unrealistic structural features of the previously proposed X-ray structure, provides an excellent description of the inelastic neutron scattering spectrum of the semi-crystalline form, and allows the correlation between microscopic structure and macroscopic properties of the polymer.

## Introduction

Progress in renewable based polymers has accelerated since they offer the possibility to reduce the environmental impact of plastics, paving the way to the UN Sustainable Development Goals (United Nations, 2020) and towards a circular economy as set forth by the European Commission (European Commission, 2015). Despite this, biobased polymers still represent today a minor fraction of all commercial polymers produced yearly (*ca*. 864 tonnes in 2021), accounting for less than 1% of the global production (European Bioplastics, 2021). A complex set of factors underlie this fact, in which economic constraints (high production costs) and their typical inferior mechanical and thermal properties have limited its market production and penetration. Nevertheless, the extensive research carried out by both academia and industry have brought new prospects with the arising of the 2,5-furandicarboxylic acid (FDCA)–a key building-block for polymers development which can impart high performance to the polymers thereof (Werpy and Petersen, 2004; Bozell and Petersen, 2010; Loos et al., 2020; de Jong et al., 2022).

**FIGURE 1:**
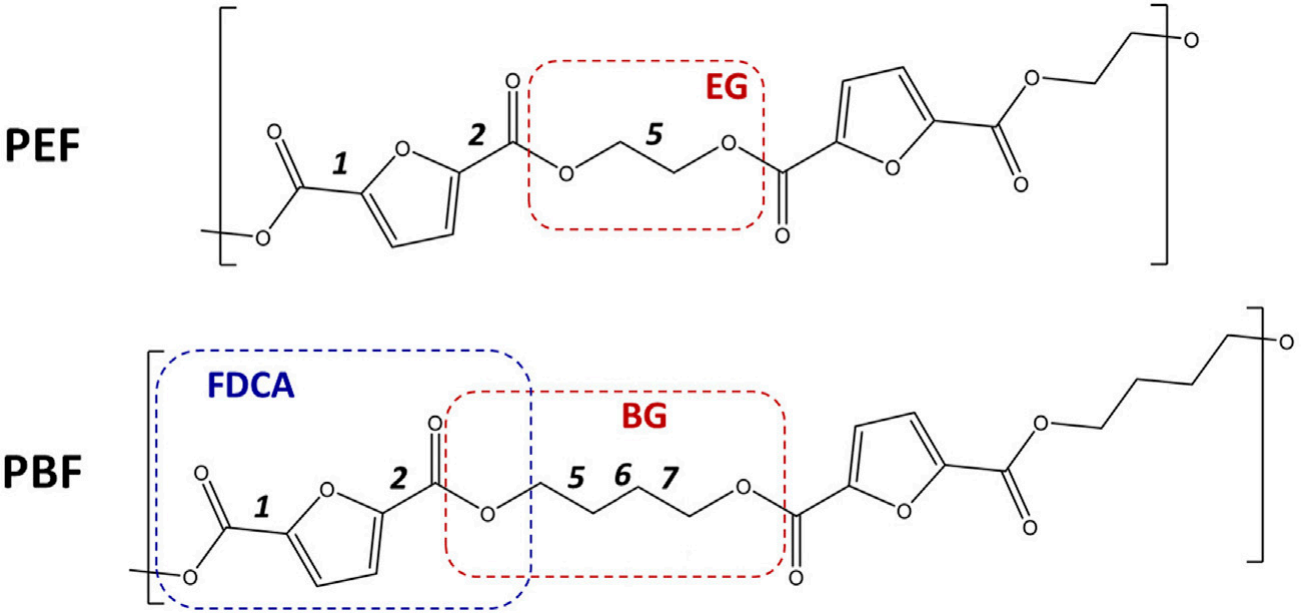
Skeletal formulas of the repeating units for poly(ethylene 2,5-furandicarboxylate) ((PEF, top) and poly(butylene 2,5-furandicarboxylate) (PBF, bottom). Dashes delimit the 1,2- and 1,4-alkyl glycol moieties—EG, BG—and the first 2,5-furandicarboxylic acid moiety–FDCA. The labels 1, 2, five to seven identify the single bonds for which rotational freedom exists. For 1 and 2, the orientation of C=O bonds relative to ring C=C bonds are described as *syn* or *anti*. For five EG and 5,6,7 BG bonds, conformations are either *trans* or *gauche.* In this way, the PBF structure shown here is described as *anti,anti-trans,trans, trans,* or *aa-ttt* for short.

Poly(ethylene 2,5-furandicarboxylate) (PEF) and poly(butylene 2,5-furandicarboxylate) (PBF)—Figures 1 – are among the most interesting FDCA-based polymers because, besides having a renewable origin and a more favorable sustainable performance, they have improved properties arising from their chemical structure. The in-depth characterization carried out so far focused mostly on PEF, due to its potential to replace the fossil-based poly(ethylene terephthalate) (PET) on packaging, *e.g*. plastic bottles (Fei et al., 2020; de Jong et al., 2022). Our group reported a vibrational spectroscopy and computational modeling study on this polymer, shedding light on important structure-property correlations (Araujo C. F. et al., 2018). A clear picture emerged, then, on how the most favorable conformational preferences both in the amorphous and crystalline regions, as well as *a* and *ß* polymorphs, are built in. Results show that, in the amorphous domains, PEF chains prefer a winding structure based on energetically favorable gauche conformation of the ethylene-glycol fragment. Yet, in the crystalline domains, polymeric chains adopt an energetically unfavorable extended *all-trans* geometry, which is stabilized by a network of C-H···O bonds linking adjacent chains. Interestingly the INS spectrum, revealing distinct low-frequency vibrational profiles for PEF and PET, confirmed the furanic “ring flipping” hindrance and stiffer polymeric chains, typically associated with enhanced O_2_, CO_2_, and H_2_O gas barrier properties (Burgess et al., 2014a; 2014c; 2014b, 2015), as well as a higher Young’s modulus. To the best of our knowledge, a similar picture has not been drawn for PBF, besides an attempt study made by (Zhu et al., 2013). Indeed, most studies focus instead on its synthesis (Carlos Morales-Huerta et al., 2016), thermal and crystallinity properties (Ma et al., 2012; Sousa et al., 2018), mechanical behavior (Zhu et al., 2013; Robles-Hernandez et al., 2020), molecular dynamics (Soccio et al., 2017; Papamokos et al., 2019; Guidotti et al., 2020; Klonos et al., 2020; Bianchi et al., 2021; Fosse et al., 2022; Poulopoulou et al., 2022) or in some of these aspects (Matos et al., 2018).

Recent publications demonstrate the high-quality simulations of the vibrational spectra of crystals using periodic boundary conditions, usually described as periodic density functional theory (DFT) calculations. Inelastic neutron scattering (INS) intensities, which are a straightforward result of the eigenvectors (atomic displacements) determined for the vibrational normal modes, are particularly well-predicted from periodic DFT, allowing the confident assignment of molecular and lattice modes in the crystal. In the case of amorphous polymer samples—or in absence of a reliable crystal structure for the crystalline domains in a semi-crystalline sample—discrete (or molecular) calculations have been used with success (Araujo C. et al., 2018; Vilela et al., 2020; Druzbicki et al., 2021; Stroupe et al., 2022). In this case, the possible polymer chain structures are simulated by resorting to short polymer fragments, typically triads. Then, the experimental spectra can be matched to a combination of proposed structures whose spectral contributions are determined from calculations. Due to the high reliability of calculations, structures that fail to adequately contribute to the experimental spectra can be excluded. This approach applies to infrared (IR) and Raman spectroscopy with some caveats, since selection rules could provide different weightings for different geometries, resulting in a non-quantitative intensity/population relationship. However, the approach is particularly suitable for INS spectroscopy, because the INS spectrum is a quantitative measurement of the vibrational density of states—i.e., it is a direct sum of contributions from the different populations—and so the INS scattering profile can be decomposed into a linear combination of contributions from dominant structural motifs (see, e.g. (Harrelson et al., 2017)). Despite the potential of the combination of these approaches they have not yet been used to describe PBF.

Further, a comparison between PEF and PBF conformational preferences and related properties was missing, apart from the obvious gain in flexibility due to the extra methylene groups of PBF. All in all, what difference does the longer alkyl chain make?

In this vein, in this work, the computational spectroscopy approach was extended to biobased PBF and a comparison with PEF is provided. By combining vibrational spectroscopy techniques—including infrared absorption (IR), Raman scattering and inelastic neutron scattering (INS)—and quantum mechanical calculations at the density functional theory (DFT) level, new insights on the structure and properties of this polymer, compared to PEF were herein achieved.

The *syn/anti* terms refer to the orientation of C=O bonds relative to the nearest ring C=C bond. In the previous work with PEF (Araujo C. F. et al., 2018), the terms s*yn/anti* were defined relative to the position of furanic oxygen atom; using the more adequate definition herein adopted simply reverses the a*nti/syn* meaning.

## Experimental details

### Synthesis of PBF

Dimethyl 2,5-furandicarboxylate (DMFDC) was synthesized following a previously reported procedure (Matos et al., 2017). Briefly, DMFDC was prepared by refluxing FDCA (5 g, 32.0 mmol) with an excess of methanol, under acidic conditions (HCl) at 80°C, for 15 h. Then, the reaction mixture was cooled down to room temperature and the resulting insoluble product was filtered off in *ca*. 70% yield.

PBF was synthesized by an adapted polytransesterification reaction procedure previously reported (Matos et al., 2018). In a first stage, DMFDC (8 g, 43.5 mmol), 1,4-butanediol (6 g, 66.5 mmol) and titanium(IV) butoxide (20 mg, 0.1 wt%) were allowed to react under a nitrogen atmosphere from 190°C up to 210°C, for 7–8 h. During the second stage, a high vacuum of 10^–3^ mbar was gradually applied, and the reaction was carried out at 210°C for 3 h. Then, the reaction was stopped and cooled down to room temperature. The polymer was purified by dissolving in a chloroform–trifluoroacetic acid mixture (6:1) and poured in an excess of methanol, filtered and dried in a vacuum oven at 40°C. The ensuing PBF was isolated in ca. 70% yield. The amorphous and semi-crystalline samples of PBF were obtained as described for PEF (Araujo C. F. et al., 2018) and characterized from powder X-ray diffraction (XRD). The XRD pattern of the amorphous sample did not reveal the presence of a crystalline fraction. The crystallinity of the semi-crystalline sample, estimated from its XRD pattern, was ca. 60%.

### X-ray diffraction

PBF samples were analyzed using X-ray diffraction studies. XRD powder patterns were collected at room temperature on a Panalytical Empyrean instrument operating with CuK*α* radiation at 40 kV and 50 mA. Samples were scanned in the 2*θ* range of 5°–70° with a step size of 0.026° and step time of 67 s.

### Vibrational spectra

PBF samples were studied using optical techniques (IR, Raman) and inelastic neutron scattering (INS). FTIR-ATR spectra were measured at room temperature using a FT Bruker IFS 55 spectrometer with a Golden Gate ATR accessory with a resolution of 2 cm^−1^. Raman spectra were collected at room temperature on a Bruker MultiRAM FT-Raman instrument with an Nd:YAG laser and using a resolution of 2 cm^−1^. The INS spectra of PBF samples were collected in the scope of project RB2000214 (Nolasco et al., 2021), using the TOSCA instrument (Parker et al., 2014; Pinna et al., 2018) at the ISIS Neutron and Muon Source of STFC’s Rutherford Appleton Laboratory (Chilton, United Kingdom) (ISIS, 2022). The samples, weighing 0.5–1 g, were placed inside a flat thin-walled aluminum can, which was then mounted perpendicular to the incident beam using a regular TOSCA centered stick. Spectra were collected below 20 K and samples were “shock-frozen” by quenching in liquid nitrogen before placement in the beam path, in order to preserve the room-temperature morphology of possible amorphous and crystalline regions. The contribution of aluminum can to the final INS spectra was found to be not negligible and has been removed by subtraction.

### Quantum chemical calculations

*Discrete (molecular) calculations:* Geometry optimizations and vibrational frequency calculations of PBF oligomers (triads, BG_3_FDCA_3_) were computed using the Gaussian 09 software, using the B3LYP density functional with the 6–311G (d,*p*) basis set. This method was found to provide a reliable description of the conformational preferences of molecular models and allows a direct comparison with previous results for PEF (Araujo C. F. et al., 2018; Papamokos et al., 2019). The initial structures were based on the well-known minima for these systems (see., e.g. (Papamokos et al., 2019)) described in Figure 1. Optimizations were performed without constraints and all the optimized structures were found to be real minima, with no imaginary frequencies. For calculated Raman and infrared spectra, vibrational frequencies were scaled by a factor of 0.967 (NIST Computational Chemistry Comparison and Benchmark Database, 2022). The inelastic neutron scattering simulated intensities were estimated from the calculated eigenvectors using the AbINS software (Dymkowski et al., 2018), a part of the Mantid package (Arnold et al., 2014). The energy values mentioned throughout the text refer to the electronic energy without zero-point correction.

*Periodic DFT calculations:* calculations were performed using the plane wave pseudopotential method as implemented in CASTEP 8.0 code (Clark et al., 2005; Refson et al., 2006). All calculations were done using the Perdew−Burke−Ernzerhof (PBE) functional based on the generalized gradient gauge (GGA) approximation (Perdew et al., 1996) supplemented with the semi-empirical dispersion correction of Tkatchenko and Scheffler (Tkatchenko and Scheffler, 2009). The plane-wave cutoff energy was set at 830 eV. Brillouin zone sampling of electronic states was performed on 2×4×5 Monkhorst−Pack grid. The initial structures were built from selected triads, considering a triclinic crystal with P1 symmetry, or obtained from (Zhu et al., 2013). Geometry optimizations were carried out with no constraints (i.e., both cell parameters and internal coordinates were relaxed) and accuracy of the optimization requested residual forces to fall below 0.005 eV A^−1^. Phonon frequencies were obtained by diagonalization of dynamical matrices calculated using density-functional perturbation theory (Milman et al., 2009). The calculated atomic displacements in each mode that are part of the CASTEP output enable visualization of the atomic motions and support the assignment of vibrational modes. The simulated inelastic neutron scattering intensities were predicted from the calculated eigenvectors using AbINS, and values were not scaled.

## Results and discussion

### Conformational landscape (from discrete molecular modelling)

The existence of a crystalline model structure is a pre-requisite to periodic DFT calculations. In the case of PBF, Zhu et al. proposed a crystal structure from fiber X-ray diffraction scans on a stretched PBF film (Zhu et al., 2013). However, the proposed structure presents several unrealistic structural features that cast serious doubts on its reliability and render it unavailing for structural characterization of the system. Among other geometrical issues, the BG alkyl chain presents C–C–C angles up to 129°, far from acceptable values for an alkyl chain*.* In the absence of doubtless crystallographic data, discrete (or molecular) calculations on polymer fragments offer a reliable alternative to assess conformational preferences of the polymer chain.

In this way, the conformational landscape of PBF was explored through DFT calculations on BG_3_FDCA_3_ triads. Due to the large number of possible combinations for this oligomer, calculations were performed for uniform conformations along the BG_3_FDCA_3_ chain. For instance, the lowest energy conformation for BG_3_FDCA_3_ was found to be *ss-gtg*, which means that all three FDCA fragments have *syn-syn* orientation and all three BG chains have *gauche-trans-gauche* conformations (see Figure 1). This “uniform chain” approach is the model expected to prompt easiest close packing of chains and, thus, to better describe the crystalline domains in the polymer. Of course, a variety of non-uniform sequences are predictable for the amorphous domains.

**FIGURE 2:**
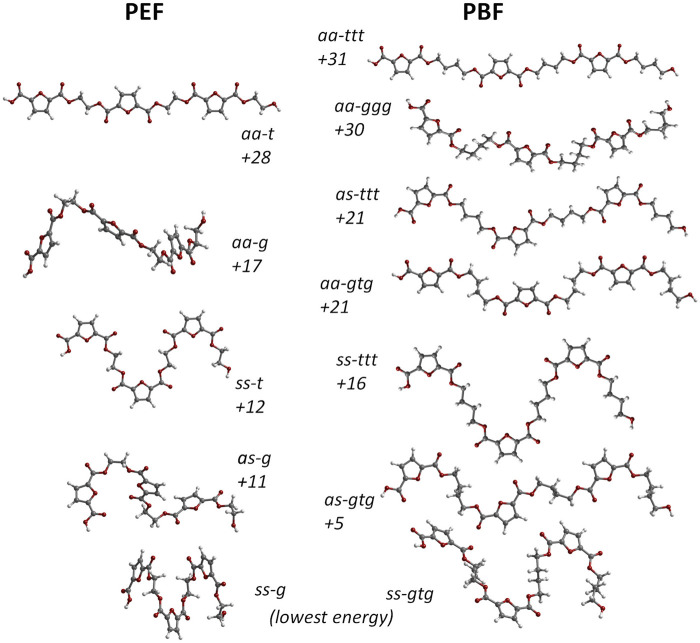
Comparison between the molecular structure of PBF triads BG_3_FDCA_3_ (right) and PEF triads EG_3_FDCA_3_ (left). The numbers indicate their relative stability expressed in terms of electronic energy, at the B3LYP/6311G (d,*p*) level, in kJ/mol. The *aa*, *as* and *ss* labels indicate the *anti-* and *syn-* orientation of the carbonyl bonds relative to nearest ring C=C bond in each FDCA fragment; for PBF, *ttt* and *gtg* indicate *trans-* and *gauche-*conformations along the BG skeleton. The *as* structures with alternate sequence *as-sa-as* for the three FDCA fragments present marginally lower energy than structures with *as-as-as* sequence and are used in this figure. Only the three *ttt* forms present a fully planar skeleton.

Figure 2 compares the structure and energies of the lower energy triads found for PEF and PBF. These triads present uniform conformations, resulting from the internal rotation around FDCA bonds labelled 1,2 and EG and BG bonds labelled 5 and 5,6,7 in Figure 1, respectively.

As in the case of PEF, in the FDCA fragment the *syn* orientation is clearly preferred over the *anti* orientation, while the alkoxy CC-CO fragments prefer the *gauche* conformation relative to the *trans* conformation. The longer alkyl chain in PBF brings an additional CC-CC torsion angle with a preference for the *trans* conformation. Hence, the lowest energy conformation in PBF is *ss-gtg* and energy raises *ca*. 30 kJ/mol up to the *aa-ggg* and *aa-ttt* conformations. The high energy *aa-ggg* structure results from the optimization of a triad extracted from the defective X-ray structure of Zhu et al., discussed above. Due to the correction of unrealistic geometrical parameters during the geometry optimization, this triad adopts the curved shape evident in Figure 2.

A noticeable feature of Figure 2 is the correlation between PBF and PEF triads and their relative energies. For instance, the lowest energy *ss-g* PEF form directly correlates with the lowest energy *ss-gtg* PBF form. In the same way, *as-g* (PEF) correlates with *as-gtg* (PBF), but the energy gap to the lowest energy form is substantially lower for PBF: 5 kJ/mol vs 11 kJ/mol. More interesting is the correlation between the “crystal-prone” (non-winding) structures in both polymers: due to the longer alkyl chain of PBF, the *aa-t* form of PEF correlates with both the *aa-ttt* and *aa-gtg* forms of PBF. And while *aa-t* and *aa-ttt* present similar energy values relative to the minima (28 and 31 kJ/mol, respectively), the *aa-gtg* form is *ca.* 7 kJ/mol below *aa-t*.

On average, the change from *gtg* to *ttt* conformation in a single BG fragment has an energy penalty of ca. 5 kJ/mol. Changes to mixed conformations, such as *gtt* or *tgg*, will require a fraction of this value. The energy penalty for a single *syn*-to-*anti* change in a single FDCA fragment falls in the range of ca. 2–3.5 kJ/mol. These values turn a large number of conformations accessible for the amorphous domain at room temperature, and, from this point of view (energetic considerations alone) also for crystalline forms. However, it is possible to discriminate the conformational preferences for crystalline and amorphous domains from a computational spectroscopy approach, as described below.

### INS spectroscopic patterns

**FIGURE 3:**
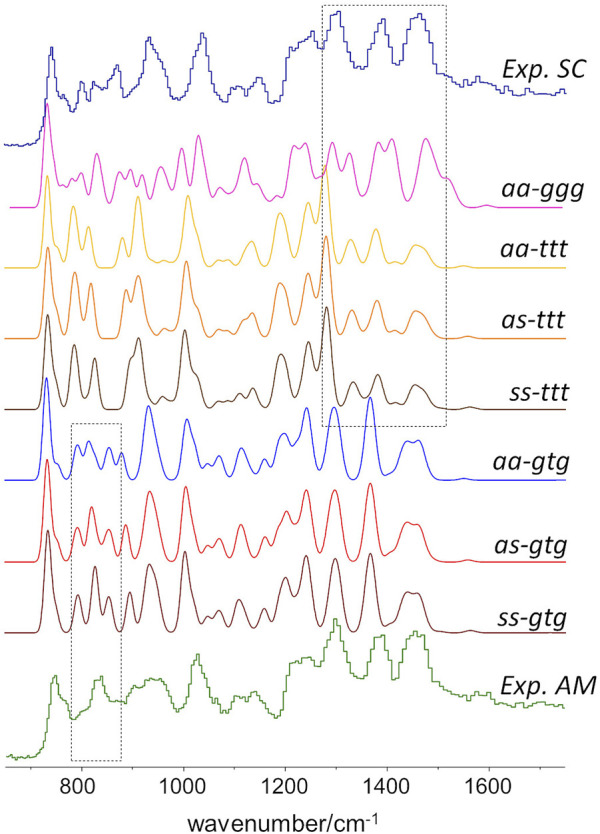
Comparison of the INS patterns in the 650–1750 cm^−1^ region theoretically predicted for the seven triads shown in Figure.2, with the experimental INS patterns of PBF semi-crystalline (top trace) and amorphous (bottom trace) samples. The dashed rectangles highlight two regions of clear pattern definition.

As stated above, since the INS intensities are relatively easy to simulate and predict, it is possible to generate reliable INS spectrum for each triad on Figure 2 and, thus, identify the INS patterns associated with each conformation. The conformations with dominant contribution to the crystalline and amorphous domains are selected from the patterns that best match the experimental spectrum of semi-crystalline and amorphous samples. Figure 3 shows the INS spectra predicted for the seven uniform triads considered, compared with the experimental INS spectra for amorphous and semi-crystalline samples.

As it can be seen, the triads bearing a BG group with *ttt* conformation fail to reproduce the intensity pattern in the ca. 1200–1450 cm^−1^ region (associated with CH_2_ bending modes). Since the intensity pattern associated with the *gtg* conformation is observed for both amorphous and semi-crystalline samples, it can be assumed that this conformation dominates both crystalline and amorphous domains. It should be mentioned that the *all-trans* (*ttt*) conformation of the alkyl chain was observed for the crystalline domains of PEF and PET, and has been reported for one polymorphic form of PBT (poly(2,5-butylene terephathalate)) (Milani and Galimberti, 2014). However, the herein described results do not support a similar behavior on PBF.

A noticeable difference between the INS spectra of amorphous and semi-crystalline samples occurs in the region of *ca*. 800–900 cm^−1^ (which embraces the out-of-plane bending of the furanic C-H bonds and the stretching of the butylene O-CH_2_ bonds). In this case, the triads combining *gtg* with *syn, syn* or *syn, anti* conformations provide a better description of the INS spectrum of the amorphous sample. The *anti,anti-gtg* triad (Figure 3, middle blue trace) relates better with the INS spectrum of the semi-crystalline sample, suggesting the predominance of this conformation in the crystalline domains, hence shedding light into the crystalline structure puzzle of PBF.

### Further details from IR and Raman spectroscopy

For PEF, it was possible to infer the conformational preferences of the crystalline and amorphous domains from optical spectroscopy, i.e., infrared and Raman spectroscopy (Araujo C. F. et al., 2018). In particular, the *trans* vs. *gauche* infrared profiles could be related with similar cases previously described (namely, from PET studies). The *anti* vs. *syn* forms of FDCA fragment were identified with support from discrete quantum mechanical frequency calculations of PEF triads.

**FIGURE 4:**
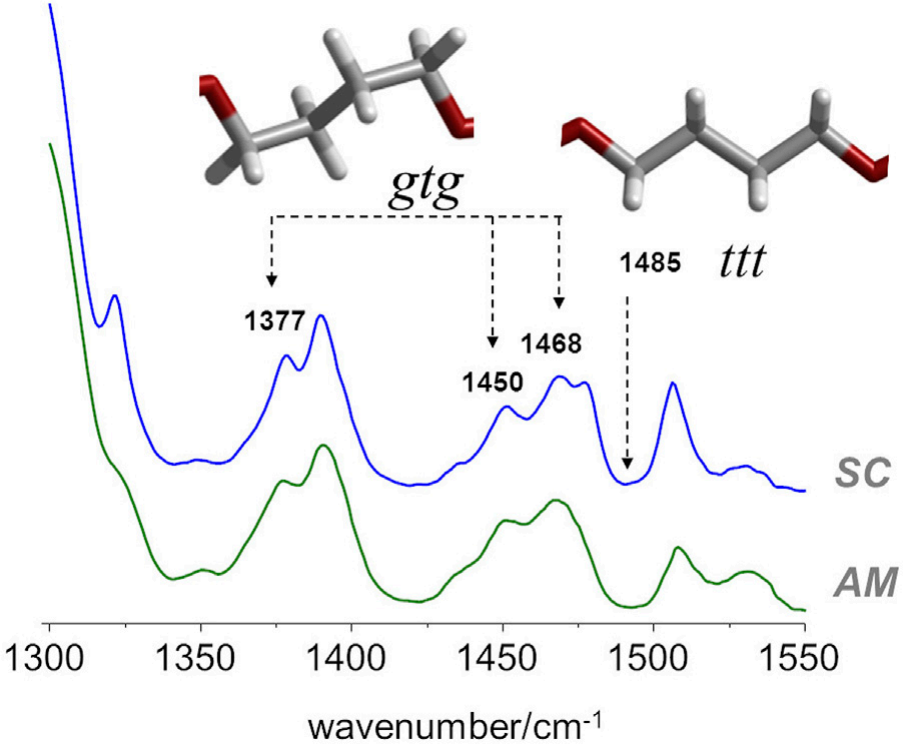
FTIR-ATR spectra of semicrystalline (top) and amorphous (bottom) samples of PBF, with identification of some bands sensitive to *gauche−trans* isomerism.

In the case of PBF, and regarding the conformation of the butylene chain, the comparison with the PBT analogue is the most straightforward. According to Milani et al. (Milani and Galimberti, 2014), there are several infrared markers associated with *gauche* and *trans* orientations of the butylene chain in PBT. For instance, the 917 cm^−1^ band is an unambiguous marker of the PBT polymorph with *gtg* chain (α polymorph) and the one at 960 cm^−1^ is a marker of the crystal form possessing chains in *all-trans* conformation (β polymorph). Other α/β markers in PBT are observed in the 1300–1550 cm^−1^ region (Milani and Galimberti, 2014). Unfortunately, in the case of PBF, the infrared spectra in these regions are not unambiguous. Nevertheless, the infrared spectra of amorphous and semi-crystalline PBF samples (Figure 4), when compared with spectra of the α/β forms of PBT in the same region, evidences a few features compatible with the prevalence of *gtg* configuration in both the crystalline and amorphous domains.

In particular, both samples present the same general profile of the *a* polymorph, with bands at 1377, 1450 and 1468 cm^−1^, and the absence of the ß polymorph marker at 1485 cm^−1^. It should be mentioned that in the case of PEF, there are large intensity changes from the amorphous to the semi-crystalline samples in this region, signaling the change from *gauche* to *trans* conformation in the BG fragment. The absence of such changes in PBF is indirect evidence of the commonness of *gtg* forms in both amorphous and crystalline domains.

**FIGURE 5:**
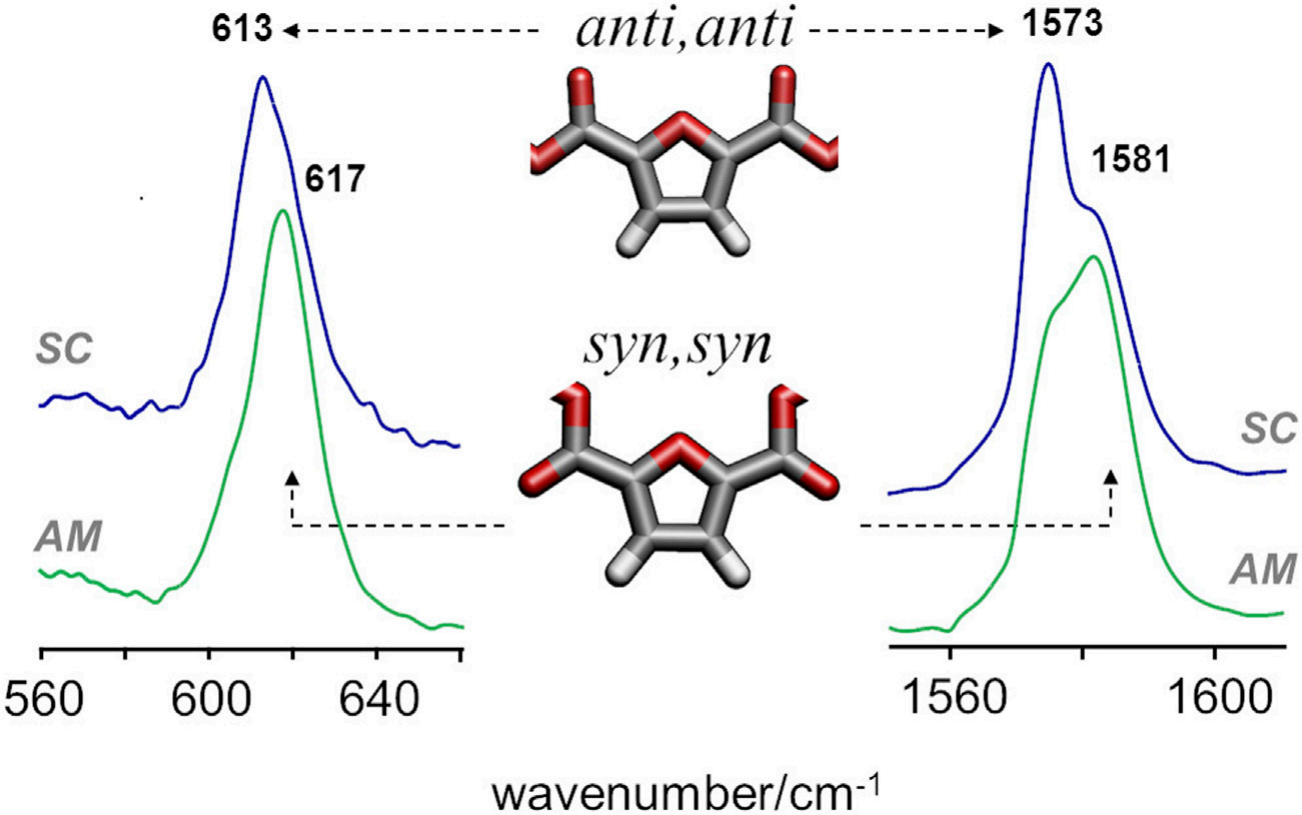
FTIR-ATR spectra of semicrystalline (top) and amorphous (bottom) samples of PBF, with identification of the bands sensitive to *syn−anti* isomerism.

A more definite conclusion can be drawn from the infrared bands associated with *syn* and *anti* conformations of the FDCA moiety. For PEF, the frequencies of two vibrational modes - ring out-of-plane deformation and C=C stretching—were found to be sensitive to the *syn/anti* conformation (Araujo C. F. et al., 2018). In PBF triads, these modes are consistently predicted to fall in the same wavenumbers and follow the same pattern, with the lowest wavenumbers associated with *anti,anti* conformation and the highest wavenumbers associated with the *syn,syn* conformation. Figure 5 compares the infrared spectra of amorphous and semi-crystalline samples in the relevant regions. The semi-crystalline sample is richer in the *anti*-FDCA forms, while the *syn*-FDCA forms are dominant in the amorphous sample.

These observations concerning the *syn-anti* conformations are consistent with the energetic profile described above and the expected changes upon crystalline packing. As observed for PEF, the *syn,syn* conformation has lower energy (and hence dominates the amorphous regions) but the *anti,anti* conformation is crucial for the establishment of C-H···O hydrogen bonds that stabilize the crystalline domains.

**FIGURE 6:**
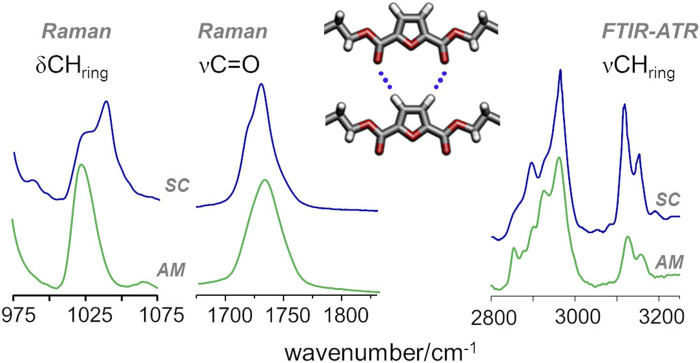
Raman and infrared spectra of amorphous (bottom) and semicrystalline (top) samples of PBF, highlighting the CH deformation (δCH_ring_) and stretching (νCH_ring_) and the C=O stretching (νC=O) vibrational modes of FDCA moieties.

In the case of PEF, the presence of C-H···O hydrogen bond interactions was inferred from a few spectroscopic changes, namely, those observed for the in-plane deformation (δ) and the stretching (ν) of furanic C−H modes and the stretching of the carbonyl C=O mode (Araujo C. F. et al., 2018). In PBF, these vibrational modes follow the same trends reported for PEF, as shown in Figure 6: from the amorphous to semicrystalline samples, the *ν*_sym_ CH_ring_ mode displays a pronounced intensification in infrared intensity, along with a red-shift from 3125 to 3118 cm^−1^, a behaviour associated with the formation of C−H···O bonds; The blue-shift of the δCH_ring_ deformation mode, clearly observed in the Raman spectra, is also a direct consequence of the restricted motion of CH_ring_ moieties due to the formation of C−H···O bonds; And the band profile of carbonyl stretching mode shows the competing effects already discussed for PEF, with a broad profile characteristic of the amorphous sample and two sharper maxima emerging with increasing crystallinity.

### Crystal structure: What can be learned from computational spectroscopy?

On the whole, what can be said about the amorphous and crystalline domains of PBF?

The amorphous domains are dominated by *gtg*-BG and *ss-*FDCA conformations, with contributions from *aa*-FDCA. “Mixed” conformations, such as *gtt-* or *ggt*-BG and *as*-FDCA forms are also probable, considering the energy balances within the amorphous polymer chain.

For the crystalline domains, the most reasonable structures steam from “extended” conformations, such as the ones observed for *aa-ttt* or *aa-gtg*. “Winding” conformations, such as those observed for the lower energy triads are not prone to crystal packing. In addition, the above mentioned observations strongly support the existence of hydrogen-bonded FDCA fragments (which requires *anti, anti* orientation of the carbonyl groups) and the prevalence of *gtg* BG chains. In this way, periodic DFT calculations were performed for a crystalline structure based on the *aa-gtg* triad. In order to have a comparison set, similar calculations were performed for two other starting structures: 1) a crystal structure built from the most linear triad (*aa-ttt*), that mimics the planar structure observed for PEF; 2) a crystal using the reported X-ray as a starting point for geometry optimization. In this last case, (and as already mentioned for discrete calculations, above) the required geometry optimization corrected the unrealistic structural parameters, and led to a geometry that can be described as *aa-ggg*.

**FIGURE 7:**
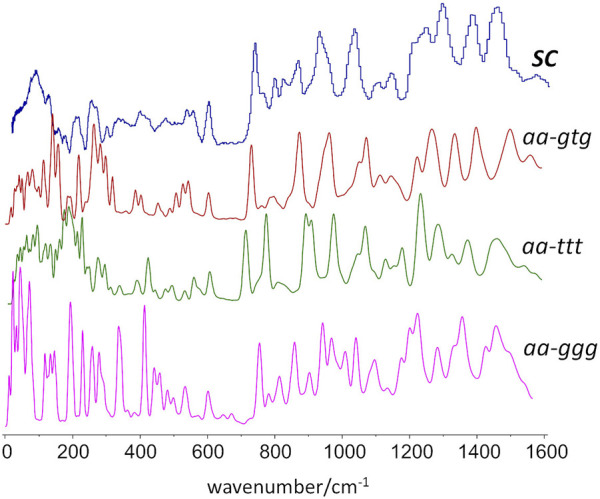
Experimental and calculated INS spectra obtained from periodic DFT calculations based on different conformational arrangements of the butylene skeleton. From top to bottom, experimental spectrum, calculated spectra for crystalline models with *gtg*, *ttt*, and *ggg* conformations.

Figure 7 compares the experimental INS spectrum of the semi-crystalline sample with the simulated INS spectra for the three crystal models. As it can be seen, the best match is provided by the periodic structure based on the *aa-gtg* conformation.

**FIGURE 8:**
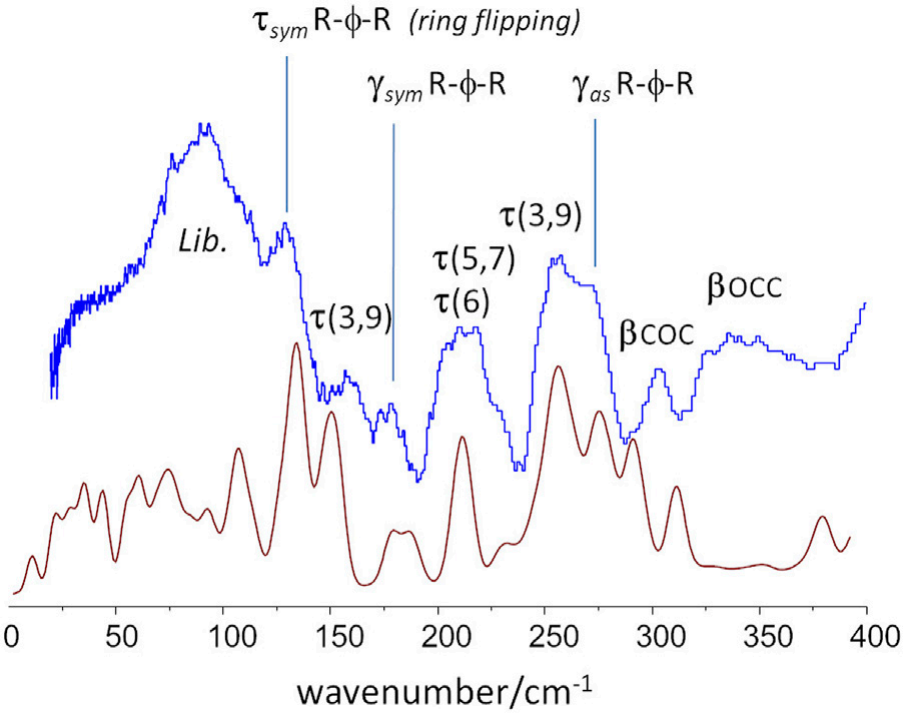
Low-wavenumber region of the INS spectra of PBF: experimental (top, blue) and calculated (bottom, red) for the *aa-gtg* crystal model.

For instance, the “intensity gap” at ca. 700 cm^−1^ was only correctly predicted for the *aa-gtg.* Above this region, the *aa-ttt* structure clearly failed to reproduce the observed intensities for the CH_2_ rock, twist, wag and scissor modes at ca. 1200–1450 cm^−1^, as already observed from discrete calculations with the molecular triad models. The *aa-ggg* spectrum produced a large number of bands in the region of 900–1200 cm^−1^, whose general profile did not match the experimental spectrum. In what concerns the 300–600 cm^−1^ interval, the bands of the experimental spectrum are nearly described by a one-to-one match to the *aa-gtg* spectrum, while both *aa-ggg* and *aa-ttt* forms deviate from this profile. Of course, below 200 cm^−1^, the intensities become increasingly “external” - or intermolecular - modes, and more dependent on crystal packing details. The description of external modes is generally known to be hampered by natural limitations of the periodic calculations (e.g. harmonic oscillator approximation, incomplete description of dispersion interactions, energy cut-offs, and the sum of numerical errors, which accumulate in the low wavenumber modes). Nevertheless, the simulated spectrum for the *aa-gtg* crystal structure provides a reasonable description of this region, allowing a reliable assignment of the low wavenumber bands, as shown in Figure 8.

This Figure 8 reveals some dynamical properties of crystalline PBF, which can be related with the longer alkyl chain of the BG fragment. For instance, the ring flipping mode, identified at *ca.* 160 cm^−1^ in PEF is observed at ca 130 cm^−1^ in PBF. This significant reduction of the barrier to rotation of the furanic ring can be ascribed to a larger flexibility of the longer BG chain in the *gtg* conformation–corroborated from the presence multiple torsional modes in the 160–260 cm^−1^ range. This larger flexibility is expected to affect the polymer properties, as discussed below.

An important vibrational mode in the comparison between 2,5-PEF and 2,4-PEF was the low-wavenumber mode described as “asymmetric out-of-plane bend of ring substituents” (Nolasco et al., 2020). In 2,4-PEF the mode was found to be sensitive to 2,4-FDCA orientation, becoming a broad band due to the random orientation of furanic rings. In the symmetrical 2,5-analogue the mode occurs as a sharp band at 272 cm^−1^, very close to the value of 270 cm^−1^ in PBF, indicating that this mode is not sensitive to the length or conformation of the alkyl chain.

How to derive macroscopic properties from the microscopic structure?

The in-depth understanding (or even prediction) of PBF macroscopic properties—namely, thermal and mechanical properties—using microscopic structure insights is the ultimate goal of the computational spectroscopy approach used in this study, and a highly desired exponent of polymer physics. However, it still remains a challenge, in large part due to the diversity of processes which ramp up into measurable properties, depending on such diverse factors such as the fine structure, chain regularity/symmetry and the chain flexibility, together with the intermolecular forces.

In addition, properties are dependent on factors such as average molecular weight, degree of crystallinity, and thermal history of the polymer sample (see, e.g. (Terzopoulou et al., 2020)).

The melting (T_m_) and the glass transition (T_g_) temperatures are thermal properties whose enthalpic component can be straightforwardly related to the intermolecular forces within the polymer bulk. While T_m_ is dependent on the interactions in the crystalline domains, T_g_ depends on cohesion of the amorphous form. Both transition temperatures are assumed to decrease with the increase of chain mobility and flexibility (Balani et al., 2015).

Crystal packing of PBF chains relies on the same intermolecular forces as PEF, namely the C-H···O contacts forming a planar layer, but the interactions between layers is hampered the gtg “ladder” motif of PBF, absent in PEF. This is in line with the T_m_ values of 211°C and 170°C reported for PEF and PBF, respectively (Burgess et al., 2014b; Guidotti et al., 2020). In what concerns the T_g_, both intermolecular forces and chain stiffness are in the equation. The presence of some C-H···O contacts, even in the amorphous form, can be assumed for both PEF and PBF. However, in PBF the longer alkyl chain reduces the probability of C-H···O contacts in the amorphous domains, thus favouring an increase in free volume and reducing chain stiffness (due to the several gauche/trans conformations available). All these effects contribute to the huge decrease of T_g_, from ca. 85°C in PEF to ca. 39°C in PBF (Burgess et al., 2014b; Guidotti et al., 2020).

The increased chain mobility of PBF relative to PEF brought by the presence of the longer alkyl chain spacer is expected to also affect properties such as gas permeation and elasticity. The gas barrier properties of PEF have been related with the restriction of the ring flipping motion (Burgess et al., 2014b; Araujo C. F. et al., 2018). As mentioned above, the ring flipping mode, identified at ca. 160 cm^−1^ in PEF is observed at ca. 130 cm^−1^ in PBF, signalling a significant reduction of the barrier to ring rotation. Hence, larger gas permeability can be predicted for PBF compared to PEF, a prediction in agreement with the recently reported values (Guidotti et al., 2020; Zhao et al., 2021).

A relevant mechanical parameter of polymeric materials due to its relevance for applications is the Young’s modulus, E, which is a measure of the elastic response to applied stress. For this property, which is more dependent on the above mentioned sample composition and processing, the experimental results are somewhat scattered. Nevertheless, a recent review (Terzopoulou et al., 2020) lists six values for PEF Young’s modulus with average value of 2.5 GPa, and twelve values for PBF Young’s modulus with average value of 1.4 GPa. Assuming a connection between the dihedral angles flexibility and polymer elasticity during the viscoelastic regime—an assumption that gets grounds on the molecular interpretation of Stirnemann (Stirnemann, 2022) for protein elasticity—the presence of a longer alkyl chain in PBF, with multiple low energy torsional modes, clearly supports the reduced stiffness of PBF relative to PEF, and, thus, in accordance with a lower PBF modulus.

## Conclusion

This work explores the conformational preferences and the structure-property correlations of biobased PBF, a longer chain analogue of PEF, from a computational spectroscopy approach, i.e., combining experimental results with computational chemistry. The approach combined infrared, Raman and inelastic neutron scattering spectroscopy with discrete and periodic density functional theory calculations, aiming at the identification of dominant structural motifs in the amorphous and crystalline regions—and, from this information at the microscopic level, predict and describe the macroscopic properties of the material.

In comparison with PEF, PBF presents higher conformational flexibility due to the presence of additional torsional degrees of freedom in the alkyl chain. Discrete calculations for triad models revealed a large number of conformations energetically accessible at room temperature, for both amorphous and crystalline forms. Nevertheless, it was possible to discriminate the conformational preferences for crystalline and amorphous domains by comparing the predicted and observed INS spectroscopic patterns and analyzing the infrared and Raman profiles in regions previously known to be sensitive to structural motifs.

The results strongly support the predominance of *gtg* conformations of the BG fragment in both the crystalline and amorphous domains. In what concerns the furandicarboxylate fragment, amorphous domains are dominated by *syn,syn* conformations, while in the crystalline domains the *anti,anti* form prevails. In addition, Raman and infrared spectra of the semi-crystalline sample unveil the spectral signature of the C-H···O hydrogen bond contacts, as found for PEF.

A possible crystalline structure, built from these conformational preferences (*aa-gtg*) and including a network of C-H···O hydrogen bond contacts, was optimized using periodic density functional theory. The *gtg* conformation of the BG fragment leads to a “ladder-like” chain. This is a relevant difference relative to PEF, for which only the fully planar *aa-t* structure allows the formation of the C-H···O hydrogen bond network. In PBF, a fully planar chain is also possible from the *ttt* conformation of the BG fragment, but with a substantially higher energy cost (ca. 10 kJ/mol per triad, as shown in Figure 2). This energy penalty is probably determinant in crystallization of PBF. Nevertheless, the existence of a higher energy PBF polymorph based on *aa-ttt* conformation cannot be discarded (and the INS main features for such *aa-ttt* polymorph are predicted from periodic DFT calculations in Figure 7).

As an ultimate goal of the computational spectroscopy approach herein described, some correlations between microscopic structure and macroscopic properties of PBF were addressed. In comparison with PEF, differences in melting and glass transition temperatures, as well as in elastic modulus and gas permeability can generally be understood at a deeper level. Furthermore, the experimental trend on lower thermal and Young’s modulus of PBF could be predicted from the increased molecular flexibility resulting from the longer alkyl chain.

## Data Availability

The datasets presented in this study can be found in online repositories. The names of the repository/repositories and accession number(s) can be found below: http://www.crystallography.net/tcod/index.php, 30000103 and 30000104; https://data.isis.stfc.ac.uk/doi/INVESTIGATION/113612429/.
